# Comparison of the Predictors of Smoking Cessation Plans between Adolescent Conventional Cigarette Smokers and E-Cigarette Smokers Using the Transtheoretical Model

**DOI:** 10.3390/children11050598

**Published:** 2024-05-15

**Authors:** Min-Hee Park, Bomi An

**Affiliations:** 1 Department of Nursing, Wonkwang University, Iksan 54538, Republic of Korea; minipark@wku.ac.kr; 2 Department of Nursing, Hannam University, Daejeon 34430, Republic of Korea

**Keywords:** adolescent, cigarette, e-cigarette, smoking cessation, vaping, transtheoretical model

## Abstract

Recently, there has been a shift in smoking patterns among adolescents, with a decrease in the prevalence of conventional cigarette smoking and an increase in the use of electronic cigarettes (e-cigarettes). The harmful effects of e-cigarettes are remarkable, highlighting the need for proactive interventions for adolescent users and smoking cessation that consider the characteristics of both conventional cigarette smokers and e-cigarette users. This study aims to investigate the smoking status of adolescent conventional cigarette and e-cigarette smokers and to analyze the predictors of their smoking cessation plans (SCPs) based on the transtheoretical model. Self-rated health, prior smoking cessation education, consciousness-raising, and dramatic relief as types of experiential processes of change, and formation of helping relationships as a type of behavioral process of change significantly differed according to the type of cigarette behavior among adolescents. The predictors of SCP among adolescents were perceived pros of smoking and academic performance among conventional cigarette smokers and behavioral process of change, perceived pros of smoking, and economic status among e-cigarette users. This study identified differences in the characteristics and predictors of SCP. Strategies tailored to each specific adolescent smoking population are further required to promote smoking cessation.

## 1. Introduction

Globally, over eight million people die from smoking annually, prompting the World Health Organization to prioritize smoking as a critical public health issue [1]. Concerted efforts to curb smoking have led to a decline in the adult smoking rate worldwide, from 32.7% in 2000 to 22.3% in 2020—about one-fourth of the global population [2]. This positive trend extends to adolescents; in the Republic of Korea, the rate of conventional cigarette smoking among adolescents has steadily decreased from 9.7% in 2013 to 4.4% in 2020, with a slight uptick to 4.5% in 2022 [3]. However, a shift in adolescent smoking patterns warrants attention, particularly as the use of conventional cigarettes declines while the prevalence of alternative smoking methods, such as e-cigarettes, increases [4]. Data from the USA-based National Youth Tobacco Survey (2011–2018) reveals a significant rise in e-cigarette use among middle and high school students, from 0.6% and 1.5% in 2011 to 2.8% and 4.9% in 2018, respectively [5]. The e-cigarette smoking rate among South Korean adolescents followed a downward trend until 2020, reaching 1.9%, but then surged to 2.9% in 2021 and 3.3% in 2022 [3].

Approximately 90% of adult smokers begin smoking before the age of 18 [6]. Therefore, smoking during adolescence is highly likely to continue into adulthood, leading to a prolonged duration of smoking and an increased risk of various health issues [7]. Although the dangers of conventional cigarette smoking are well documented, recent studies have begun to unveil the harmful effects of e-cigarettes. Kumar et al. [8] reported that e-cigarette aerosols cause inflammation and are associated with a higher risk of lung cancer and respiratory infections [9]. Additionally, the Centers for Disease Control and Prevention [10] reported that, as of February 2020, there were 2807 cases of lung diseases attributed to e-cigarette use in the United States, further emphasizing the risks of e-cigarettes.

Moreover, studies have shown that the likelihood of transitioning to conventional cigarette smoking is more than fourfold for those who have used e-cigarettes [11], suggesting that adolescents who use e-cigarettes are also at potential risk for the hazards associated with conventional cigarette smoking. Given the undeniable risks of e-cigarettes, there is a pressing need for targeted smoking cessation interventions for adolescents. However, most of the existing programs focus primarily on conventional cigarettes, with only a limited number of programs specifically and exclusively addressing e-cigarettes [12].

Smoking cessation plans (SCPs) are characterized by a well-defined and strong intention to quit smoking, indicating a readiness to engage in smoking cessation behaviors [13]. To effectively guide adolescent smokers toward successful smoking cessation, it is crucial to first establish their intention to quit, which serves as the foundation for SCPs. This concept aligns with the transtheoretical model (TTM) proposed by Prochaska and DeClemente [14], which outlines the stages of change in health-related behaviors as follows: (1) Precontemplation (no intention to change behavior within the next six months); (2) Contemplation (intention to change behavior within the next six months); (3) Preparation (intention to take action within the next month or having attempted behavior change several times in the past year); (4) Action (having made lifestyle changes in the past six months); and (5) Maintenance (maintaining the new behavior for more than six months without reverting to previous behaviors), (6) Termination (having no temptation) [15]. The TTM is based on the understanding that health behavior changes occur in stages, and it advocates for tailored interventions that match an individual’s readiness or stage. This model has been applied in various health behavior studies [16,17] and has informed research on adolescent smoking cessation. Studies have emphasized the need for personalized intervention strategies based on individuals’ intentions and readiness to quit smoking [18,19]. Meta-analyses of smoking cessation studies utilizing the TTM have further confirmed that interventions considering the stages of change are more effective than those that do not [16]. However, to the best of our knowledge, there is a gap in research analyzing smoking cessation intentions among adolescents based on their smoking patterns using the TTM.

### Aims of the Study

Against this backdrop, this study aims to examine the smoking status of adolescents who smoke conventional cigarettes and those who use e-cigarettes and to identify the predictors of their SCPs based on the TTM. The specific objectives are as follows: (1) Understand the differences in general characteristics and smoking characteristics between adolescent conventional cigarette smokers and e-cigarette users; (2) Examine the differences in processes of change, decisional balance, self-efficacy, and SCPs between adolescent conventional cigarette smokers and e-cigarette users; and (3) Compare the predictors of SCPs between adolescent conventional cigarette smokers and e-cigarette users.

## 2. Materials and Methods

### 2.1. Study Design

This study adopted a descriptive survey to compare the predictors of SCPs between adolescent conventional cigarette smokers and e-cigarette users.

### 2.2. Participants

Adolescent smokers were invited to enroll in the study with the cooperation of the smoking cessation support centers in 17 metropolitan and provincial regions of the Republic of Korea. The enrolled participants were current adolescent smokers registered at smoking cessation support centers and provided informed consent to participate in the study.

The sample size was calculated using G*power 3.1. Regarding a previous study [20], analysis for an odds ratio (OR) of 1.5, a significance level of 0.05, and a power of 0.80 required a minimum sample size of 208. To account for dropouts, 300 participants were surveyed, and after excluding adolescents who have not smoked a conventional or e-cigarette in the past 30 days, a total of 237 were included in the analysis.

### 2.3. Instruments

#### 2.3.1. Conventional Cigarette Smoking and E-Cigarette Use

The participants of this study were classified according to their response to the question, “Indicate the type of tobacco you have smoked at least once in the past 30 days”. Adolescents who chose “conventional cigarettes” were categorized as “conventional cigarette smokers”, while those who indicated “liquid e-cigarettes” or “heat-not-burn e-cigarettes” were classified as “e-cigarette users”. Dual users who smoke “conventional cigarettes” and “liquid e-cigarettes” or “heat-not-burn e-cigarettes” were classified as “conventional cigarette smokers”.

#### 2.3.2. SCPs

SCPs were determined based on the response to whether there is a plan to quit smoking in the future. Those who answered that they plan to quit within one month or six months were classified as “having an SCP”, while those who responded that they plan to quit someday but not within the next six months or have no current plan to quit were classified as “not having an SCP”.

#### 2.3.3. General Characteristics

Participants’ general characteristics surveyed were age, sex, school level, economic status, academic performance, living arrangement, father’s education level, mother’s education level, self-rated health (SRH), current alcohol use, vigorous physical activity, breakfast consumption, and perceived stress.

Sex was classified as male or female, and the school level was divided into “middle school” and “high school”. Academic performance and family economic status were categorized as “average or above” and “below average”. Living arrangements were classified as “living with parents” or “not living with parents”, and parental education level was divided into “high school or lower”, “college or higher”, and “unknown”. SRH was assessed with the question, “How do you think about your health status in general?” and the responses were classified as “healthy”, “average”, or “unhealthy”. Drinking was assessed using the question, “How many days in the past 30 days have you had at least one drink?” and the responses were classified as “non-drinker” for no drinking in the past 30 days and “drinker” for drinking at least once a month. Vigorous physical activity was assessed with the question, “How many days in the past seven days have you engaged in vigorous physical activity for at least 20 min that made you sweat or breathe hard?” and classified as “yes” for three or more days per week and “no” for fewer than three days per week. Breakfast consumption was assessed with the question, “How many days in the past seven days have you had breakfast (excluding only milk or juice consumption)?” and the responses were categorized as “no” for 0–4 times and “yes” for 5 times or more. Perceived stress was assessed using the question, “How much stress do you usually feel?” The responses feeling a great deal of stress, feeling a lot of stress, or feeling some stress were considered “yes”, while not feeling much stress or not feeling any stress was considered “no”.

#### 2.3.4. Smoking Characteristics

The smoking characteristics surveyed included age at smoking initiation, family smoking, friends’ smoking, prior smoking cessation education, and awareness of smoking cessation campaigns.

Age at smoking initiation was assessed with the questions, “When did you first try conventional cigarettes (even just a puff or two)?”; “When did you first use a liquid e-cigarette?”; and “When did you first use a heat-not-burn e-cigarette?” and responses were recorded as the starting age. Family smoking was determined by asking participants to indicate if any family members currently smoke, and responses were classified as “no” if no one was indicated and “yes” if at least one family member was reported as a smoker. Friends’ smoking was assessed with the question, “Do any of your close friends smoke?” and responses were divided into “almost none smoke” and “most smoke”. Prior smoking cessation education was classified based on whether participants had received smoking prevention and cessation education at school in the last 12 months. Awareness of smoking cessation campaigns was classified based on whether participants had seen or heard any smoking cessation-related promotions in the last 12 months.

#### 2.3.5. Variables of the TTM

For variables of the TTM, the process of change in smoking cessation (experiential process of change, behavioral process of change), decisional balance for smoking (perceived pros and cons of smoking), and self-efficacy were examined.

The process of change in smoking cessation refers to the adaptive mechanisms used to change one’s smoking behavior to cessation. Using the simplified tool developed by Prochaska and DiClemente [14], five experiential processes of change (consciousness raising, dramatic relief, environmental reevaluation, self-reevaluation, social liberation) were measured with ten items, and five behavioral processes of change (counter conditioning, helping relationships, reinforcement management, self-liberation, stimulus control) were measured with ten items. Frequent experience or action related to smoking cessation in the past month was rated as 5, and no such experience or action was rated as 1. A higher score indicates a greater application of that process of change. The score ranges from 10–50 for the experiential process of change and 10–50 for the behavioral process of change. The Cronbach’s alpha was 0.86 at the time of development and 0.89 and 0.88, respectively, in this study.

The decisional balance for smoking is a variable that determines the level of decision-making involved in smoking, consisting of an individual’s perceived pros and cons of smoking. We used the Smoking Decisional Balance Scale developed by Velicer, DiClemente, Prochaska, and Brandenburg [21] to measure this factor. This tool consists of 10 items related to the pros of smoking and 10 items related to the cons of smoking. Each item is rated on a scale from 1 “not important at all”, to 5 “very important”, and a higher score indicates higher perceived pros or cons of smoking. The Cronbach’s alpha was 0.87 for the pros of smoking and 0.90 for the cons of smoking at the time of tool development, and 0.90 in this study.

Self-efficacy refers to the smoker’s ability to refrain from smoking in various situations, and it was measured using nine items developed by Velicer, DiClemente, Rossi, and Prochaska [22] for smoking cessation self-efficacy. Each item was rated on a scale from 1 “not confident at all” to 5 “very confident”. The total score ranges from 5–45, and a higher score indicates higher self-efficacy. The Cronbach’s alpha was 0.98 at the time of development and 0.93 in this study.

### 2.4. Data Collection and Ethical Considerations

Data were collected via an online survey from September 2022 to February 2023 through 17 smoking cessation support centers nationwide. Before data collection, this study was approved by the Institutional Review Board (IRB) at W University (WKIRB-202208-SB-068), and ethical considerations were regarded at all steps of the study. The purpose and content of this study were communicated to the staff of smoking cessation support centers in 17 regions nationwide via official letters and emails, and the data collection procedure was explained over the phone. Data were collected only when both guardians and adolescents voluntarily agreed to participate in the study. The study information page explained the purpose and content of the study, assurance of confidentiality and lack of harm, freedom to withdraw from the study at any time, and freedom to refuse to answer any question. After completing the survey, participants were provided with an online coupon as a token of appreciation. The collected data were anonymized and coded for computer processing and will be disposed of after three years.

### 2.5. Data Analysis

Data were analyzed using SPSS Statistics version 26, and statistical significance was determined at a level of 0.05. The following statistical analyses were performed: First, adolescents’ general characteristics were analyzed, and the differences between the general characteristics and smoking characteristics according to smoking patterns were analyzed using χ^2^ test or an independent sample *t*-test. Second, differences in the process of change, decisional balance, and self-efficacy according to smoking patterns were analyzed with an independent sample *t*-test. Third, the predictors of SCPs among adolescent conventional cigarette smokers and e-cigarette users were analyzed with binomial logistic regression analysis.

## 3. Results

### 3.1. General Characteristics according to Smoking Patterns

SRH (χ^2^ = 9.98, *p* = 0.007) and prior smoking cessation education (χ^2^ = 7.48, *p* = 0.006) significantly differed according to smoking patterns. Although 57.3% of adolescent conventional cigarette smokers considered themselves healthy, only 38.3% of adolescent e-cigarette users considered themselves healthy, indicating that adolescents who smoke conventional cigarettes perceive themselves as healthier. Furthermore, prior smoking cessation education was reported by 62.5% of adolescent conventional cigarette smokers and 78.7% of adolescent e-cigarette users, indicating that e-cigarette users had relatively more experience with smoking cessation education (Table 1).

### 3.2. Process of Change, Decisional Balance, Self-Efficacy, and SCPs according to Smoking Patterns

We analyzed the differences in the experiential process of change, the behavioral process of change, the pros and cons of smoking, and self-efficacy according to smoking patterns. Only consciousness-raising (t = −2.67, *p* = 0.008) and dramatic relief (t = −2.49, *p* = 0.014) of the experiential process of change and the formation of helping relationships of the behavioral process of change (t = −2.24, *p* = 0.026) significantly differed according to smoking patterns (Table 2).

### 3.3. Predictors of SCPs

In the univariate logistic regression analysis, experiential process of change (OR = 2.62, *p* = 0.016), behavioral process of change (OR = 3.05, *p* = 0.003), pros of smoking (OR = 0.39, *p* = 0.010), self-efficacy (OR = 1.86, *p* = 0.015), sex (OR = 2.62, *p* = 0.027), academic performance (OR = 2.51, *p* = 0.032), and family’s smoking (OR = 0.33, *p* = 0.030) were determined as significant predictors of SCP in adolescent conventional cigarette smokers. The significant predictors were analyzed using a multivariate logistic regression analysis. The results showed that the odds of having SCPs were significantly lower with increasing perceived pros of smoking (OR = 0.25, *p* = 0.009) and significantly higher with good academic performance (OR = 4.60, *p* = 0.008) (Table 3).

For adolescent e-cigarette users, univariate logistic regression revealed that experiential process of change (OR = 2.64, *p* < 0.001), behavioral process of change (OR = 3.69, *p* < 0.001), pros of smoking (OR = 0.57, *p* = 0.039), self-efficacy (OR = 1.97, *p* = 0.001), and economic status (OR = 3.11, *p* = 0.021) are significant predictors of SCPs. These significant predictors were analyzed using multivariate logistic regression. The results indicated that the odds of having SCPs were significantly higher with an increasing behavioral process of change (OR = 3.85, *p* = 0.024) and better economic status (OR = 3.78, *p* = 0.017) and significantly lower with an increased perception of the pros of smoking (OR = 0.43, *p* = 0.015) (Table 4).

## 4. Discussion

### 4.1. Findings

This study aimed to determine the predictors of SCPs among adolescent conventional cigarette smokers and adolescent e-cigarette users based on the TTM, ultimately providing foundational data for interventions tailored to the smoking characteristics of adolescents.

The analysis revealed that adolescent e-cigarette users perceive themselves as less healthy and have a higher rate of receiving smoking cessation education compared to their counterparts who smoke conventional cigarettes. However, there is a paucity of prior studies comparing these two groups of adolescent smokers, which limits the ability to make direct comparisons with existing literature. E-cigarette users tend to believe that e-cigarettes are less harmful [23] and safer [24] than conventional cigarettes, irrespective of their past smoking history. This belief is a major reason for the use of e-cigarettes among adolescents [25]. Moreover, most e-cigarette users perceive that they can control the adverse effects of e-cigarettes and can easily quit before becoming addicted. Consequently, they often use e-cigarettes as a transitional step toward achieving their goal of quitting conventional cigarettes [23]. Considering these characteristics, the findings of this study suggest that adolescents who perceive their health as poor or who have received smoking cessation education are more likely to use e-cigarettes as an alternative to conventional cigarettes, possibly due to health concerns. The aforementioned perception regarding e-cigarettes is believed to have contributed to the sustained increase in e-cigarette use among adolescents worldwide. Given that e-cigarettes contain nicotine, there should also be consideration for the potential issue of addiction to e-cigarettes.

Regarding smoking patterns, adolescent e-cigarette users were found to have significantly higher rates of experiencing consciousness-raising and dramatic relief during the experiential process of change, as well as a helping relationship among the behavioral process of change, compared to adolescent conventional cigarette smokers. Consciousness-raising and dramatic relief are primarily utilized during the transition from the pre-contemplation to the contemplation stage in the stages of change [14], and these stages involve the process of recognizing the need for behavior change and experiencing emotions related to the behavior to acknowledge the issue [15]. The helping relationship represents social support for adolescents toward changing their smoking behavior [15] and considering that adolescent smoking behavior is significantly influenced by peers [26], it is a crucial process in altering adolescent smoking behavior. The study findings reveal that the use of e-cigarettes is more prevalent among adolescents who have a heightened awareness of smoking-related issues and have access to a social support system for smoking cessation. Moreover, the overall scores for the experiential process of change and the behavioral process of change were generally higher in the e-cigarette user group. In light of these results, as well as previous findings that adolescents perceive e-cigarettes as an aid for smoking cessation [27,28] and consider e-cigarettes as less harmful than conventional cigarettes [29], it appears that adolescents who have undergone the experiential process of change and the behavioral process of change utilize e-cigarettes as part of their efforts to quit smoking or to avoid exposure to harmful substances from conventional cigarette smoking. Nonetheless, the absence of prior studies comparing the differences in the experiential process of change and the behavioral process of change between e-cigarette and conventional cigarette users limits the interpretation of the results; thus, further research is needed on this topic.

Regarding the predictors of SCPs in the two smoker groups, an increased perception of the pros of smoking negatively impacted the likelihood of having an SCP. This aligns with existing findings that the perceived pros of smoking outweigh barriers in individuals at the pre-contemplation stage [15], which is also supported by other studies [30,31]. For adolescent conventional cigarette smokers, better academic performance was associated with a higher likelihood of having a SCP. This is supported by previous studies indicating that higher academic performance is correlated with a greater probability of successful smoking cessation [32] and that poor academic performance is positively correlated with smoking [33]. Goodman and Capitman [34] reported that a lower GPA was identified as a predictor of moderate to heavy smoking, further validating our results. Programs targeting adolescents should strategically focus on correcting misconceptions about the pros of smoking to encourage smoking cessation behavior.

Among adolescent e-cigarette users, the likelihood of having a SCP was higher for those who experienced the behavioral process of change, that is, those who have implemented behavioral coping strategies to modify their behaviors. Generally, the experiential process is known to influence the early stages of change, such as pre-contemplation and contemplation, while the behavioral process impacts the action and maintenance stages of change [14]. This has been demonstrated by previous studies applying the TTM to smoking behavior [35,36]. In this study, we anticipated that the experiential process would be a significant factor influencing the likelihood of having an SCP, as the participants were in the early stages of change, including pre-contemplation, contemplation, and preparation. However, the findings revealed that the behavioral process had a more substantial impact, contradicting the previous findings. In a study on adolescents, Kim et al. [18] showed significant differences in the smoking behavioral process of change between individuals at the pre-contemplation and contemplation stages, indicating that the behavioral process of change is a more critical factor than the experiential process of change in explaining adolescent smoking behavior. Similarly, studies by Rios et al. [19] describe behavioral processes as important concomitants in the stage of change in smoking cessation behavior, which is in line with our findings. These findings suggest that behavioral change is crucial in formulating a SCP for adolescents using e-cigarettes, and intervention programs should consider incorporating this as a primary strategy. However, since there are some variations in the results of previous studies, further research is needed to validate these findings.

Another predictor of SCPs among adolescent e-cigarette users was household economic status, where the odds of having an SCP were higher with higher household economic status. Economic status is a classic indicator of socioeconomic status (SES), and in general, studies on smoking in adults have shown that lower SES is associated with a failure to quit smoking [37]. However, a systematic review on predictors of adolescent smoking cessation [38] indicated that high SES is “probably unrelated” to smoking cessation, inconsistent with our findings. Research on the relationship between adolescent e-cigarette use and SES is currently limited, and findings are inconsistent. Simon et al. [39] found a relationship between SES and past-month e-cigarette use, whereas Barrington-Trimis et al. [40] and Moore et al. [41] found no relationship between SES and past-month or lifetime e-cigarette use, respectively. Because it is difficult to assess SES accurately among adolescents compared to adults, the association between SES and smoking tends to be unclear [42]. Additional analysis is needed to clarify the association between SES and e-cigarette cessation among adolescents.

### 4.2. Limitations

This study had a few limitations. First, the data were collected through self-report surveys that rely on the participants’ memory, thus posing the possibility of recall biases. Second, the cross-sectional design only confirms associations between variables and cannot establish causality. Third, considering that many adolescent e-cigarette users also use other types of tobacco [43], we included e-cigarette users who also smoke conventional cigarettes. Further research is needed on adolescents who exclusively use e-cigarettes. Finally, although we recruited participants from smoking cessation support centers across 17 metropolitan and provincial regions of South Korea, the sample size was limited, and most smoking adolescents are not registered at smoking cessation support centers. Thus, the findings of this study cannot be generalized to the entire adolescent population in Korea, necessitating further validation of the study.

## 5. Conclusions

In reflection of the recent trend of adolescents transitioning from conventional cigarette smoking to e-cigarette use, this study compared the characteristics and predictors of SCPs between adolescent conventional cigarette smokers and e-cigarette users. Significant differences were observed in SRH, prior smoking cessation education, consciousness-raising, and dramatic relief in the experiential process of change and helping relationships in the behavioral process of change according to smoking patterns. Perceived pros of smoking and academic performance were identified as significant predictors among adolescent conventional cigarette smokers, while the behavioral process of change, perceived pros of smoking, and economic status were identified as significant predictors among adolescent e-cigarette users. This study underscores the need for differentiated strategies based on smoking patterns to effectively promote smoking cessation behavior among adolescents.

Given the limited availability of smoking cessation programs specifically targeting adolescent e-cigarette users, there is a pressing need for the development of tailored programs based on a comparative analysis between conventional cigarette smokers and e-cigarette users. This study is significant in that it applies the TTM that is widely used in health behavior intervention programs to provide foundational data for establishing intervention strategies tailored to the smoking type among adolescents. As there is currently limited research on the predictors of SCPs among adolescents according to their smoking patterns using the TTM, further follow-up studies are recommended to establish tailored smoking cessation intervention strategies based on smoking patterns.

## Figures and Tables

**Table 1:** General and smoking characteristics according to smoking patterns.

Variable	Category	Total	Smoking Patterns		χ^2^/t (*p*)
			Conventional Cigarette	E-Cigarette	
General characteristics					
Age		17.49 ± 1.60	17.41 ± 1.96	17.55 ± 1.32	−0.69 (0.490)
Sex	Male	142 (59.9)	61 (63.5)	81 (57.4)	0.88 (0.347)
	Female	95 (40.1)	35 (36.5)	60 (42.6)	
School level	Middle school	45 (19.0)	13 (13.5)	32 (22.7)	3.11 (0.078)
	High school	192 (81.0)	83 (86.5)	109 (77.3)	
Academic performance	Average or higher	112 (47.3)	51 (53.1)	61 (43.3)	2.23 (0.135)
	Below average	125 (52.7)	45 (46.9)	80 (56.7)	
Economic status	Average or higher	203 (85.7)	85 (88.5)	118 (83.7)	1.10 (0.295)
	Below average	34 (14.3)	11 (11.5)	23 (16.3)	
Living arrangement	Living with parents	187 (78.9)	75 (78.1)	112 (79.4)	0.06 (0.809)
	Not living with parents	50 (21.1)	21 (21.9)	29 (20.6)	
Father’s education	High school or lower	88 (37.1)	39 (40.6)	49 (34.8)	0.90 (0.637)
	College or higher	87 (36.7)	34 (35.4)	53 (37.6)	
	Don’t know	62 (26.2)	23 (24.0)	39 (27.7)	
Mother’s education	High school or lower	81 (34.2)	29 (30.2)	52 (36.9)	1.25 (0.534)
	College or higher	93 (39.2)	41 (42.7)	52 (36.9)	
	Don’t know	63 (26.6)	26 (27.1)	37 (26.2)	
SRH	Healthy	109 (46.0)	55 (57.3)	54 (38.3)	9.98 (0.007)
	Average	93 (39.2)	33 (34.4)	60 (42.6)	
	Unhealthy	35 (14.8)	8 (8.3)	27 (19.1)	
Drinking	Non-drinker	91 (38.4)	43 (44.8)	48 (34.0)	2.79 (0.095)
	Drinker	146 (61.6)	53 (55.2)	93 (66.0)	
Vigorous physical activity	No	179 (75.5)	74 (77.1)	105 (74.5)	0.21 (0.646)
	Yes	58 (24.5)	22 (22.9)	36 (25.5)	
Breakfast	No	178 (75.1)	73 (76.0)	105 (74.5)	0.08 (0.783)
	Yes	59 (24.9)	23 (24.0)	36 (25.5)	
Stress	No	140 (59.1)	62 (64.6)	78 (55.3)	2.03 (0.154)
	Yes	97 (40.9)	34 (35.4)	63 (44.7)	
Smoking characteristics					
Age at smoking initiation		14.13 ± 1.70	14.40 ± 1.80	13.95 ± 1.62	1.95 (0.053)
Family’s smoking	No	66 (27.8)	31 (32.3)	35 (24.8)	1.67 (0.434)
	Yes	151 (63.7)	58 (60.4)	93 (66.0)	
	Don’t know	20 (8.4)	7 (7.3)	13 (9.2)	
Friends’ smoking	Almost none smoke	74 (31.2)	33 (34.4)	41 (29.1)	0.75 (0.388)
	Most smoke	163 (68.8)	63 (65.6)	100 (70.9)	
Prior smoking cessation education	No	66 (27.8)	36 (37.5)	30 (21.3)	7.48 (0.006)
	Yes	171 (72.2)	60 (62.5)	111 (78.7)	
Awareness of smoking cessation campaigns	No	83 (35.0)	32 (33.3)	51 (36.2)	0.20 (0.653)
	Yes	154 (65.0)	64 (66.7)	90 (63.8)	

**Table 2:** Process of change, decisional balance, self-efficacy, and smoking cessation plans according to smoking patterns.

Variable		Total	Smoking Patterns		χ^2^/t (*p*)
			Conventional Cigarette	E-Cigarette	
Experiential process of change		2.87 ± 0.73	2.77 ± 0.60	2.94 ± 0.80	−1.73 (0.085)
Consciousness-raising		3.00 ± 0.93	2.82 ± 0.75	3.12 ± 1.02	−2.67 (0.008)
Dramatic relief		2.89 ± 0.87	2.72 ± 0.73	3.01 ± 0.94	−2.49 (0.014)
Environmental re-evaluation		2.92 ± 0.89	2.88 ± 0.74	2.95 ± 0.99	−0.62 (0.533)
Self-re-evaluation		2.69 ± 0.90	2.61 ± 0.81	2.74 ± 0.96	−1.10 (0.270)
Social liberation		2.84 ± 0.87	2.81 ± 0.79	2.85 ± 0.93	−0.36 (0.716)
Behavioral process of change		2.78 ± 0.72	2.73 ± 0.67	2.81 ± 0.75	−0.83 (0.406)
Counter conditioning		2.74 ± 0.83	2.72 ± 0.79	2.75 ± 0.86	−0.25 (0.801)
Helping relationship		3.00 ± 0.96	2.83 ± 0.89	3.11 ± 0.99	−2.24 (0.026)
Reinforcement management		2.68 ± 0.91	2.64 ± 0.85	2.71 ± 0.96	−0.57 (0.572)
Self-liberation		3.03 ± 0.96	2.99 ± 0.93	3.06 ± 0.98	−0.53 (0.598)
Stimulus control		2.46 ± 0.97	2.48 ± 0.90	2.44 ± 1.03	0.37 (0.709)
Pros of smoking		2.98 ± 0.68	2.98 ± 0.69	2.98 ± 0.67	−0.01 (0.991)
Cons of smoking		3.21 ± 0.65	3.17 ± 0.66	3.25 ± 0.64	−0.95 (0.345)
Self-efficacy		2.79 ± 0.95	2.82 ± 0.92	2.77 ± 0.97	0.35 (0.730)
Smoking cessation plans	Yes	133 (56.1)	58 (60.4)	75 (53.2)	1.21 (0.271)
	No	104 (43.9)	38 (39.6)	66 (46.8)	

**Table 3:** Predictors of smoking cessation plans among adolescent conventional cigarette smokers.

Variable	Crude Model		Adjusted Model	
	OR (95% CI)	*p*	OR (95% CI)	*p*
Experiential process of change	2.62 (1.20~5.73)	0.016	0.57 (0.08~4.01)	0.575
Behavioral process of change	3.05 (1.47~6.30)	0.003	4.25 (0.77~23.47)	0.097
Pros of smoking	0.39 (0.19~0.79)	0.010	0.25 (0.09~0.71)	0.009
Cons of smoking	0.67 (0.35~1.29)	0.230		
Self-efficacy	1.86 (1.13~3.06)	0.015	1.49 (0.71~3.12)	0.287
Sex (male)	2.62 (1.11~6.19)	0.027	2.06 (0.68~6.27)	0.202
Economic status (average or higher)	0.13 (0.02~1.06)	0.057		
Academic performance (average or higher)	2.51 (1.08~5.81)	0.032	4.60 (1.50~14.11)	0.008
Living arrangement (Living with parents)	0.92 (0.34~2.50)	0.875		
School level (high school)	0.95 (0.29~3.15)	0.929		
Father’s education (≥college or higher)	1.67 (0.63~4.43)	0.303		
Father’s education (Don’t know)	0.64 (0.23~1.80)	0.395		
Mother’s education (≥college or higher)	0.91 (0.34~2.47)	0.857		
Mother’s education (don’t know)	0.53 (0.18~1.56)	0.246		
SRH (healthy)	2.06 (0.46~9.18)	0.345		
SRH (average)	1.06 (0.23~4.98)	0.939		
Drinking (drinker)	0.58 (0.25~1.35)	0.207		
Vigorous physical activity (Yes)	0.93 (0.35~2.45)	0.885		
Breakfast (Yes)	0.81 (0.31~2.09)	0.662		
Stress (Yes)	1.09 (0.46~2.57)	0.841		
Age at smoking initiation	1.18 (0.93~1.50)	0.166		
Family’s smoking (Yes)	0.33 (0.12~0.90)	0.030	0.53 (0.17~1.67)	0.280
Family’s smoking (Don’t know)	0.22 (0.04~1.22)	0.083	0.38 (0.05~2.73)	0.339
Friends’ smoking (Most)	0.99 (0.42~2.34)	0.978		
Prior smoking cessation education (yes)	1.15 (0.49~2.67)	0.747		
Awareness of smoking cessation campaigns (yes)	1.07 (0.45~2.54)	0.883		

Note: Reference categories for categorical variables were as follows: stages of changes in smoking cessation behaviors (no plans), sex (female), economic status (below average), academic performance (below average), living arrangement (not living with parents), school level (middle school), parents’ education (high school or lower), SRH (unhealthy), drinking (non-drinker), vigorous physical activity (no), breakfast (no), stress (no), family’s smoking (no), friends’ smoking (almost none), prior smoking cessation education (no), awareness of smoking cessation campaigns (no).

**Table 4:** Predictors of smoking cessation plans among adolescent e-cigarette users.

Variable	Crude Model		Adjusted Model	
	OR (95% CI)	*p*	OR (95% CI)	*p*
Experiential process of change	2.64 (1.57~4.43)	<0.001	0.99 (0.35~2.78)	0.982
Behavioral process of change	3.69 (2.01~6.78)	<0.001	3.85 (1.19~12.43)	0.024
Pros of smoking	0.57 (0.34~0.97)	0.039	0.43 (0.22~0.85)	0.015
Cons of smoking	1.47 (0.86~2.50)	0.158		
Self-efficacy	1.97 (1.33~2.91)	0.001	1.44 (0.92~2.25)	0.109
Sex (male)	1.40 (0.72~2.75)	0.320		
Economic status (average or higher)	3.11 (1.19~8.12)	0.021	3.78 (1.26~11.32)	0.017
Academic performance (average or higher)	1.70 (0.87~3.35)	0.122		
Living arrangement (Living with parents)	1.82 (0.80~4.17)	0.156		
School level (high school)	1.63 (0.74~3.62)	0.226		
Father’s education (≥college or higher)	0.77 (0.35~1.69)	0.519		
Father’s education (Don’t know)	0.59 (0.25~1.38)	0.225		
Mother’s education (≥college or higher)	0.58 (0.27~1.26)	0.170		
Mother’s education (don’t know)	0.80 (0.34~1.87)	0.601		
SRH (healthy)	2.12 (0.83~5.43)	0.116		
SRH (average)	1.17 (0.47~2.91)	0.737		
Drinking (drinker)	0.83 (0.41~1.67)	0.601		
Vigorous physical activity (Yes)	1.14 (0.53~2.43)	0.742		
Breakfast (Yes)	1.32 (0.62~2.84)	0.474		
Stress (Yes)	1.06 (0.54~2.06)	0.868		
Age at smoking initiation	1.14 (0.92~1.41)	0.224		
Family’s smoking (Yes)	0.71 (0.32~1.57)	0.397		
Family’s smoking (Don’t know)	0.57 (0.16~2.06)	0.393		
Friends’ smoking (Most)	0.97 (0.47~2.02)	0.943		
Prior smoking cessation education (yes)	1.65 (0.73~3.73)	0.225		
Awareness of smoking cessation campaigns (yes)	1.67 (0.83~3.33)	0.148		

Note: Reference categories for categorical variables: stages of changes in smoking cessation behaviors (no plans), sex (female), economic status (below average), academic performance (below average), living arrangement (not living with parents), school level (middle school), parents’ education (high school or lower), SRH (unhealthy), drinking (non-drinker), vigorous physical activity (no), breakfast (no), stress (no), family’s smoking (no), friends’ smoking (almost none), prior smoking cessation education (no), awareness of smoking cessation campaigns (no).

## Data Availability

The datasets are available on request due to privacy protection concerns; access to the research data is limited to the principal investigator and co-investigators, as explained to all participants.

## References

[1] World Health Organization Fact Sheet: Tobacco. https://www.who.int/news-room/fact-sheets/detail/tobacco.

[2] World Health Organization WHO Report on the Global Tobacco Epidemic 2021: Addressing New and Emerging Products. https://www.who.int/publications/i/item/9789240032095.

[3] Ministry of Education Announcement of the Results of the 2022 Student Health Examination and Youth Health Behavior Survey. https://www.moe.go.kr/boardCnts/viewRenew.do?boardID=294&boardSeq=94695&lev=0&m=020.

[4] Ma C., Xi B., Li Z., Wu H., Zhao M., Liang Y., Bovet P. (2021). Prevalence and trends in tobacco use among adolescents aged 13–15 years in 143 countries, 1999–2018: Findings from the Global Youth Tobacco Surveys. Lancet Child Adolesc. Health.

[5] Wang T.W., Gentzke A., Sharapova S., Cullen K.A., Ambrose B.K., Jamal A. (2018). Tobacco product use among middle and high school students—United States, 2011–2017. MMWR Morb. Mortal. Wkly. Rep..

[6] US Department of Health and Human Services The Health Consequences of Smoking—50 Years of Progress: A Report of the Surgeon General. https://aahb.org/Resources/Pictures/Meetings/2014-Charleston/PPT%20Presentations/Sunday%20Welcome/Abrams.AAHB.3.13.v1.o.pdf.

[7] Wilson N., Battistich V., Syme S.L., Boyce W.T. (2002). Does elementary school alcohol, tobacco, and marijuana use increase middle school risk?. J. Adolesc. Health.

[8] Kumar P., Geisinger M., DeLong H.R., Lipman R.D., Araujo M.W.B. (2020). Living under a cloud: Electronic cigarettes and the dental patient. J. Am. Dent. Assoc..

[9] Znyk M., Jurewicz J., Kaleta D. (2021). Exposure to heated tobacco products and adverse health effects, a systematic review. Int. J. Environ. Res. Public Health.

[10] Centers for Disease Control and Prevention Outbreak of Lung Injury Associated with the Use of E-Cigarette or Vaping, Products. https://www.cdc.gov/tobacco/basic_information/e-cigarettes/severe-lung-disease.html.

[11] O’Brien D., Long J., Quigley J., Lee C., McCarthy A., Kavanagh P. (2021). Association between electronic cigarette use and tobacco cigarette smoking initiation in adolescents: A systematic review and meta-analysis. BMC Public Health.

[12] Liu J., Gaiha S.M., Halpern-Felsher B. (2020). A breath of knowledge: Overview of current adolescent e-cigarette prevention and cessation programs. Curr. Addict. Rep..

[13] Noar S.M., Zimmerman R.S. (2005). Health Behavior Theory and cumulative knowledge regarding health behaviors: Are we moving in the right direction?. Health Educ. Res..

[14] Prochaska J.O., Diclemente C.C. (1983). Stages and processes of self-change of smoking: Toward an integrative model of change. J. Consult. Clin. Psychol..

[15] Velicer W.F., Prochaska J.O., Fava J.L., Norman G.J., Redding C.A. (1998). Detailed overview of the transtheoretical model. Homeostasis.

[16] Spencer L., Pagell F., Hallion M.E., Adams T.B. (2002). Applying the transtheoretical model to tobacco cessation and prevention: A review of literature. Am. J. Health Promot..

[17] Glanz K., Rimer B.K., Viswanath K. (2015). Health Behavior: Theory, Research, and Practice.

[18] Kim Y.H. (2006). Adolescents’ smoking behavior and its relationships with psychological constructs based on transtheoretical model: A cross-sectional survey. Int. J. Nurs. Stud..

[19] Rios L.E., Herval Á.M., Ferreira R.C., Freire M.D.C.M. (2019). Prevalences of stages of change for smoking cessation in adolescents and associated factors: Systematic review and meta-analysis. J. Adolesc. Health.

[20] Jung S., Shin S.R. (2021). Factors affecting stages of change in smoking cessation and intention to quit among Asian students in Korea based on the transtheoretical model. Korean J. Adult Nurs..

[21] Velicer W.F., Diclemente C.C., Prochaska J.O., Brandenburg N. (1985). Decisional balance measure for assessing and predicting smoking status. J. Pers. Soc. Psychol..

[22] Velicer W.F., Diclemente C.C., Rossi J.S., Prochaska J.O. (1990). Relapse situations and self-efficacy: An integrative model. Addict. Behav..

[23] Ambrose B.K., Rostron B.L., Johnson S.E., Portnoy D.B., Apelberg B.J., Kaufman A.R., Choiniere C.J. (2014). Perceptions of the relative harm of cigarettes and e-cigarettes among U.S. youth. Am. J. Prev. Med..

[24] Gorukanti A., Delucchi K., Ling P., Fisher-Travis R., Halpern-Felsher B. (2017). Adolescents’ attitudes towards e-cigarette ingredients, safety, addictive properties, social norms, and regulation. Prev. Med..

[25] Tsai J., Walton K., Coleman B.N., Sharapova S.R., Johnson S.E., Kennedy S.M., Caraballo R.S. (2018). Reasons for electronic cigarette use among middle and high school students–National Youth Tobacco Survey, United States, 2016. MMWR Morb. Mortal. Wkly. Rep..

[26] Ahuja N., Kedia S.K., Jiang Y., Xie L., Ward K.D., Pichon L.C., Dillon P.J., Yu X. (2022). Factors associated with E-cigarette quitting behavior among adolescents in the United States: A prospective observational study. J. Adolesc. Health.

[27] Bold K.W., Kong G., Cavallo D.A., Camenga D.R., Krishnan-Sarin S. (2016). Reasons for trying e-cigarettes and risk of continued use. Pediatrics.

[28] Lee J.A., Lee S., Cho H.J. (2017). The relation between frequency of e-cigarette use and frequency and intensity of cigarette smoking among South Korean adolescents. Int. J. Environ. Res. Public Health.

[29] Anand V., McGinty K.L., O’Brien K., Guenthner G., Hahn E., Martin C.A. (2015). E-cigarette use and beliefs among urban public high school students in North Carolina. J. Adolesc. Health.

[30] Ham O.K. (2007). Stages and processes of smoking cessation among adolescents. West. J. Nurs. Res..

[31] Kim Y., Kang S., Vongjaturapat N. (2023). Application of transtheoretical model to explain adolescents’ smoking behavior. J. Subst. Use.

[32] Mertens A.E.J., Kunst A.E., Lorant V., Alves J., Rimpelä A., Clancy L., Kuipers M.A.G. (2021). Smoking cessation among adolescents in Europe: The role of school policy and programmes. Drug Alcohol Depend..

[33] Kinnunen J.M., Paakkari L., Rimpelä A.H., Kulmala M., Richter M., Kuipers M.A.G., Kunst A.E., Lindfors P.L. (2022). The role of health literacy in the association between academic performance and substance use. Eur. J. Public Health.

[34] Goodman E., Capitman J. (2000). Depressive symptoms and cigarette smoking among teens. Pediatrics.

[35] Ahmad A., Singh J. (2022). Influence of processes of change on stages of change for smoking cessation. J. Appl. Soc. Sci..

[36] Ham O.K., Lee Y.J. (2007). Use of the transtheoretical model to predict stages of smoking cessation in Korean adolescents. J. Sch. Health.

[37] Cambron C., Lam C.Y., Cinciripini P., Li L., Wetter D.W. (2020). Socioeconomic status, social context, and smoking lapse during a quit attempt: An ecological momentary assessment study. Ann. Behav. Med..

[38] Vallata A., O’Loughlin J., Cengelli S., Alla F. (2021). Predictors of cigarette smoking cessation in adolescents: A systematic review. J. Adolesc. Health.

[39] Simon P., Camenga D.R., Kong G., Connell C.M., Morean M.E., Cavallo D.A., Krishnan-Sarin S. (2017). Youth e-cigarette, blunt, and other tobacco use profiles: Does SES matter?. Tob. Reg. Sci..

[40] Barrington-Trimis J.L., Berhane K., Unger J.B., Cruz T.B., Huh J., Leventhal A.M., Urman R., Wang K., Howland S., Gilreath T.D. (2015). Psychosocial factors associated with adolescent electronic cigarette and cigarette use. Pediatrics.

[41] Moore G., Hewitt G., Evans J., Littlecott H.J., Holliday J., Ahmed N., Moore L., Murphy S., Fletcher A. (2015). Electronic-cigarette use among young people in Wales: Evidence from two cross-sectional surveys. BMJ Open.

[42] Hiscock R., Bauld L., Amos A., Fidler J.A., Munafò M. (2012). Socioeconomic status and smoking: A review. Ann. N. Y Acad. Sci..

[43] McCabe S.E., West B.T., Veliz P., Boyd C.J. (2017). E-cigarette use, cigarette smoking, dual use, and problem behaviors among U.S. adolescents: Results from a national survey. J. Adolesc. Health.

